# A Five-Helix-Based SARS-CoV-2 Fusion Inhibitor Targeting Heptad Repeat 2 Domain against SARS-CoV-2 and Its Variants of Concern

**DOI:** 10.3390/v14030597

**Published:** 2022-03-13

**Authors:** Lixiao Xing, Xinfeng Xu, Wei Xu, Zezhong Liu, Xin Shen, Jie Zhou, Ling Xu, Jing Pu, Chan Yang, Yuan Huang, Lu Lu, Shibo Jiang, Shuwen Liu

**Affiliations:** 1 Key Laboratory of Medical Molecular Virology (MOE/NHC/CAMS), School of Basic Medical Sciences, Shanghai Frontiers Science Center of Pathogenic Microbes and Infection, Shanghai Institute of Infectious Disease and Biosecurity, Fudan University, Shanghai 200032, China; 20111010065@fudan.edu.cn (L.X.); xuwei11@fudan.edu.cn (W.X.); 17111010065@fudan.edu.cn (Z.L.); 18301050164@fudan.edu.cn (X.S.); 19211010046@fudan.edu.cn (J.Z.); 21111010081@m.fudan.edu.cn (L.X.); 17111010015@fudan.edu.cn (J.P.); 2 State Key Laboratory of Organ Failure Research, Guangdong Provincial Key Laboratory of New Drug Screening, School of Pharmaceutical Sciences, Southern Medical University, Guangzhou 510515, China; xinfengxu848@163.com (X.X.); virus6522@smu.edu.cn (C.Y.); huangyuan@smu.edu.cn (Y.H.)

**Keywords:** SARS-CoV-2, variant of concern, fusion inhibitor, 5-Helix, six-helix bundle, heptad repeat 1, heptad repeat 2

## Abstract

The prolonged duration of the severe acute respiratory syndrome coronavirus-2 (SARS-CoV-2) pandemic has resulted in the continuous emergence of variants of concern (VOC, e.g., Omicron) and variants of interest (VOI, e.g., Lambda). These variants have challenged the protective efficacy of current COVID-19 vaccines, thus calling for the development of novel therapeutics against SARS-CoV-2 and its VOCs. Here, we constructed a novel fusion inhibitor-based recombinant protein, denoted as 5-Helix, consisting of three heptad repeat 1 (HR1) and two heptad repeat 2 (HR2) fragments. The 5-Helix interacted with the HR2 domain of the viral S2 subunit, the most conserved region in spike (S) protein, to block homologous six-helix bundle (6-HB) formation between viral HR1 and HR2 domains and, hence, viral S-mediated cell–cell fusion. The 5-Helix potently inhibited infection by pseudotyped SARS-CoV-2 and its VOCs, including Delta and Omicron variants. The 5-Helix also inhibited infection by authentic SARS-CoV-2 wild-type (nCoV-SH01) strain and its Delta variant. Collectively, our findings suggest that 5-Helix can be further developed as either a therapeutic or prophylactic to treat and prevent infection by SARS-CoV-2 and its variants.

## 1. Introduction

Coronavirus disease 2019 (COVID-19), caused by SARS-CoV-2, has posed an unprecedented threat to public health, with over 5.67 million deaths and 370 million confirmed diseases through 29 January 2021 (https://covid19.who.int/, accessed on 29 January 2021). Thus far, vaccination has proven its efficacy in disease prophylaxis and control. However, the continuing worldwide spread and transmission of SARS-CoV-2 have resulted in the emergence of many variants of concern (VOC) and interest (VOI), as defined by WHO (https://www.who.int/en/activities/tracking-SARS-CoV-2-variants/, accessed on 29 January 2021). The spike (S) mutations in variants, including K417N/T, E484K and N501Y, have changed antigenic properties resulting in the evasion of the immune protection induced by vaccines [1]. The newly emerged VOC Omicron (B.1.1.529), which contains 32 mutations within its S protein, has exhibited significantly increased resistance to the neutralizing antibodies elicited by the current COVID-19 vaccines and therapeutics [2,3], thus calling for the development of more effective and broader-spectrum antivirals against these VOCs and VOIs.

**Figure 1:**
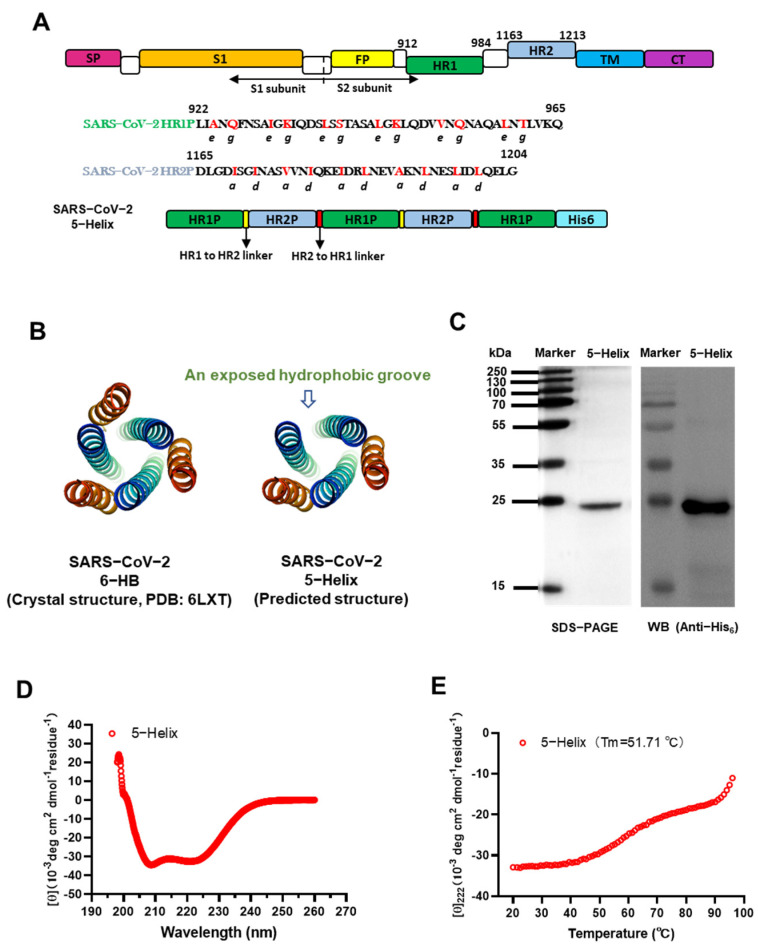
Design and detection of 5-Helix protein based on HR1 and HR2 sequences of the SARS-CoV-2 S protein S2 subunit. (**A**) Schematic representation of SARS-CoV-2 S protein and 5-Helix protein. Three copies of HR1 fragment (L922 to Q965, green) and two copies of HR2 fragment (D1165 to G1204, cyan) were sequentially linked with flexible linkers, including the GGSGG linker between HR1 and HR2 linker (yellow) and GSSGG linker between HR2 and HR1 linker (red). SP, signal peptide; FP, fusion peptide; HR1, heptad repeat 1; HR2, heptad repeat 2; TM, transmembrane domain; CT, cytoplasmic tail. (**B**) Predicted structure of 5-Helix and its comparison with the crystal structure of 6-HB formed by the HR1 peptide (cyan) and HR2 peptide (orange) of the SARS-CoV-2 S protein (PDB entry 6LXT, adapted from [7]). (**C**) Verification of expressed and purified 5-Helix by SDS-PAGE and Western blotting assays. The bacterial lysate was immunoblotted with HRP-labeled anti-His_6_ antibody; band of the purified 5-Helix was revealed in the SDS-PAGE gel stained with Coomassie blue. (**D**) The secondary structure of 5-Helix in PBS (pH = 7.4) was examined by CD spectroscopy. Double minima at 208 nm and 222 nm were revealed. (**E**) Thermal unfolding of 5-Helix. The melting curve and its melting temperature (*T_m_*) are shown.

The functional domains, known as heptad repeat 1 (HR1) and 2 (HR2) in the S2 subunit of the SARS-CoV-2 spike (S) protein, play critical roles in forming the six-helix bundle (6-HB) fusion core (Figure 1A,B) and mediating viral fusion and entry into the host cell. HR1 and HR2 also have the most conserved sequences in S protein. Consequently, these domains are important targets for the development of viral fusion/entry inhibitors [4]. In response, we previously developed a series of HR1-targeting SARS-CoV-2 fusion inhibitory peptides, such as EK1 [5], HR2P [6], EK1C4 [7] and EKL1C [8]. However, no peptide targeting HR2 of the SARS-CoV-2 S protein has been reported so far, even though HR2 has a more conserved sequence than HR1 (Figure S1) [6]. Previous studies on the peptides derived from the HIV-1 gp41 NHR and CHR (also known as HR1 and HR2) peptides have demonstrated that NHR peptides, such as N36, exhibit antiviral activity 2 to 3 orders lower than that of CHR peptides, such as C34. Compared to a CHR peptide, an NHR peptide generally contains more hydrophilic amino acids to form NHR-trimer with the concomitant propensity to aggregate in physiological solutions [9]. Extrapolating, it is entirely possible that peptides derived from the SARS-CoV-2 HR1 domain [6] may behave in a manner similar to that of HIV-1 gp41 NHR peptides [9,10]. 

Therefore, in this study, we designed and constructed a plasmid encoding the SARS-CoV-2 5-Helix protein consisting of three HR1 and two HR2 portions derived from the HR1 and HR2 regions of the S2 subunit of SARS-CoV-2 (Figure 1A). In a competition experiment, we found that 5-Helix could interact with the SARS-CoV-2 HR2 peptide to form 6-HB, thus potently inhibiting 6-HB formation between HR1 and HR2 of SARS-CoV-2 and, hence, S-mediated cell–cell fusion and infection by SARS-CoV-2 and its variants of concern, including the recently emerged Omicron. These findings suggest that 5-Helix is a promising candidate for further development as either a therapeutic or prophylactic to treat or prevent infection by current and future SARS-CoV-2 variants.

## 2. Materials and Methods

### 2.1. Cells, Viruses and Peptides

293T, Huh 7, Caco-2 and Calu-3 cells were obtained from the American Type Culture Collection (ATCC). They were cultured in Dulbecco’s Modified Eagle’s Medium (DMEM) with 10% fetal bovine serum (FBS), 100 U/mL penicillin and 100 μg/mL streptomycin in a humidified incubator containing 5% CO_2_ at 37 °C.

Authentic SARS-CoV-2 WT strain (nCoV-SH01) and Delta variant (B.1.617.2) were maintained in the BSL-3 Laboratory of the Shanghai Medical College, Fudan University [8]. The gene sequence of the Delta variant was confirmed by next-generation sequencing. 

SARS-CoV-2 HR1 and HR2 peptide (sequences shown in Figure 1A), C-terminal biotinylated HR2 peptide and HIV-1 gp41 HR2 peptide (LLEQENKEQQNQSEEILSHILSTYNNIERDWEMW) were synthesized by Synpeptide Co., Ltd. (Nanjing, China).

### 2.2. Expression and Purification of 5-Helix

The 5-Helix gene was codon-optimized, synthesized and subcloned into pET-28a (+) vector (Huada Genomics, Beijing, China) for expression in *Escherichia coli* strain BL21 (DE3) (Invitrogen). The engineered bacteria were cultured in LB medium containing 50 μg/mL of kanamycin at 37 °C to an optical density (OD_600_) of 0.5 induced with 1 mM isopropyl-β-D-thiogalactopyranoside (IPTG) at 22 °C for 8 h. The harvested bacteria were resuspended in phosphate-buffered saline (PBS) containing 10 mM imidazole and 500 mM NaCl and subsequently lysed by ultrasonication on ice. The lysate was centrifuged and purified by Ni-NTA (Qiagen, Hilden, Germany). The purified protein was dialyzed against PBS to remove imidazole and further confirmed by SDS-PAGE and Western blot using an anti-His_6_ antibody (Figure 1A, 5-Helix containing a His_6_-tag), as reported previously [11].

### 2.3. Circular Dichroism Spectroscopy Analysis

The secondary structure of 5-Helix was examined by circular dichroism (CD) spectroscopy. Purified protein was diluted in PBS to a final concentration of 10 μM. CD spectral data from 198 nm to 260 nm were measured on a spectropolarimeter (Model J-715, Jasco Inc., Tokyo, Japan) at 25 °C with 0.1 nm resolution, 0.1 cm path length, 5.0 nm bandwidth, 4.0 s response time and a 50 nm/min scanning speed. Thermal denaturation was monitored at 222 nm in temperatures ranging from 20 °C to 100 °C at the rate of 1 °C/min. Melting temperature (*T_m_*), the heat required to melt a complex, or unfold or denature a protein, which determines the thermostability (the ability of a protein to maintain structural and functional properties when heated) [12], was calculated by Jasco software as described previously [13].

### 2.4. Cell–Cell Fusion Assay

5-Helix-mediated inhibitory activity on SARS-CoV-2 S-mediated cell–cell fusion was assessed as previously described [5]. Briefly, 293T cells were transfected with pAAV-IRES-EGFP vector plasmid encoding SARS-CoV-2 S protein as effector cells. Twelve hours later, freshly trypsinized 293T/SARS-CoV-2-S/GFP cells were mixed with an equal volume of three-fold serially diluted 5-Helix at concentrations ranging from 10,000 nM to 14 nM, or PBS, in wells of a flat-bottom 96-well plate at 37 °C for 2 h. After incubation, the mixture of effector cells and protein, or PBS, was gently transferred to the plate seeded with ACE2 receptor-expressing Huh 7 cells at 37 °C for 2 h. Finally, the percentage of fused cells was counted under a fluorescence microscope (Zeiss, Jena, Germany), and the 50% inhibitory concentration was calculated.

### 2.5. Inhibition of Pseudotyped Coronavirus Infection

Plasmids of pNL4-3.luc.RE (the luciferase reporter-expressing HIV-1 backbone) and pcDNA3.1 encoding SARS-CoV-2-S were co-transfected with 293T cells using VigoFect (Vigorous Biotechnology, Beijing, China) [8]. The spike gene of SARS-CoV-2 wildtype strain and variants were obtained from the GISAID Platform (https://platform.gisaid.org, accessed on 29 January 2021, SARS-CoV-2 (IIUM316), GenBank: MW079429.1; Alpha (CUMC-21081002): OM757358.1; Beta (TUS-Siena7): OM736179.1; Gamma (AR-UFRO-0018): OM146072.1; Delta (CA-CDPH-500041690): OM754056.1; Omicron (CA-LACPHL-AF06801): OM757978.1; Lambda (MA-UMASSMED-P026A04): OM486769.1). The supernatant containing the released pseudotyped coronavirus pseudovirus (PsV) was harvested and centrifuged at 3000× *g* for 10 min and preserved at -80 °C. In order to determine TCID_50_ of these PsVs, it was initially diluted two-fold and followed by three-fold serial dilution in a 96-well plate and then transferred to Caco-2 cells. Cells were re-fed in a fresh medium after 12 h. The luminescence detection was performed at 48 h post-infection. A luminescence value ten-fold higher than the untreated cell control was considered as positive [14]. TCID_50_ was determined by using the Reed-Muench method. In order to examine the inhibitory activity of 5-Helix against coronavirus PsV infection, Caco-2 cells were seeded in a 96-well plate (1 × 10^4^ cells per well). Twelve hours later, 100 TCID_50_ of coronavirus PsV were mixed with an equal volume of 5-Helix, which was serially diluted with PBS at 37 °C for 30 min. The mixture was transferred to the Caco-2 cells. After 12 h, cells were re-fed with fresh medium, followed by luminescence measurement after 48 h incubation.

### 2.6. Inhibition of Authentic Coronavirus Infection

Inhibitory activity against authentic coronavirus infection was conducted in a Biosafety Level 3 (BSL-3) laboratory of Fudan University. An inhibition assay was performed as described previously [8]. Briefly, Calu-3 cells were seeded into a 96-well plate. Twenty-four hours later, 0.01 multiplicity of infection (MOI) authentic SARS-CoV-2 was mixed with an equal volume of 5-Helix, which was serially diluted with PBS at 37 °C for 30 min. The mixture was transferred to the Calu-3 cells. Forty-eight hours later, the supernatants were collected for quantification of SARS-CoV-2 copies. After extraction of viral RNA, reverse-transcription quantitative PCR (RT-qPCR) was used to test SARS-CoV-2 N gene copies. The primers and probe for detection of the SARS-CoV-2 N gene mRNA were as follows: forward primer: 5′-GGGGAACTTCTCCTGCTAGAAT-3′; reverse primer: 5′-CAGACATTTTGCTCTCAAGCTG-3′; and probe: 5′-FAM-TTGCTGCTGCTTGACAG ATTTAMRA-3′. Then, qPCR was performed on the Mastercycler ep realplex Real-time PCR System (Eppendorf) with the One-Step PrimeScrip RT-PCR Kit (Takara, Tokyo, Japan) according to the manufacturer’s instructions.

### 2.7. Measurement of Cytotoxicity

The 5-Helix cytotoxicity to Huh 7 cells, Caco-2 cells and Calu-3 cells was measured by using the Cell Counting Kit-8 (CCK-8; Dojindo, Kumamoto, Japan). In brief, cells of each cell line were seeded in a 96-well plate (1 × 10^4^ cells per well), respectively. Twelve hours later, the cell medium was replaced by DMEM serially diluted protein or peptide. Forty-eight hours later, ten-fold diluted CCK-8 solution was added and then incubated for 4 h. Finally, the absorbance was measured at 450 nm.

### 2.8. Solid-Phase Binding ELISA

In a binding assay, 0.1 μg/mL (50 μL) 5-Helix protein was coated on a 96-well half-area plate (Costar, New York, NY, USA) at 4 °C overnight. The coated plates were then blocked with 3 % non-fat milk at 37 °C for 2 h. Then, serially diluted biotinylated SARS-CoV-2 HR2P peptide or HIV-1 HR2 peptide was pipetted into each well for 1 h. Bound HR2 peptide was detected by horseradish peroxidase (HRP)-labeled Streptavidin (Beyotime Biotechnology, Beijing, China). The absorbance at 450 nm was detected by an Infinite M200 reader (Tecan, Männedorf, Sweden). In a separate binding assay, the wells of a plate were coated with 2 μg/mL Streptavidin (Sangon Biotechnology, Shanghai, China). After blocking, biotinylated SARS-CoV-2 or HIV-1 HR2P peptides were added. After washes, serially diluted 5-Helix was added. Finally, the bound 5-Helix was detected by using HRP-conjugated anti-His_6_ antibody (Proteintech, Wuhan, China), and the absorbance at 450 nm was detected as described above [15].

### 2.9. Biolayer Interferometry

Real-time binding kinetics between 5-Helix and biotinylated SARS-CoV-2 HR2P was recorded on an Octet QK system (Fortebio, San Francisco, CA, USA) at 25 °C. Biotinylated SARS-CoV-2 HR2P was loaded onto a streptavidin biosensor. Serially diluted 5-Helix was associated with HR2P for 1200 s, followed by another 1200 s dissociation time in PBS containing 0.02% Tween. The equilibrium dissociation constant (K_D_) was calculated by Octet QK software by a 1:1 binding model [16].

### 2.10. Native Polyacrylamide Gel Electrophoresis (N-PAGE)

Serially diluted 5-Helix (2-fold dilution, with the highest final concentration of 128 μM) was incubated with an equal volume of SARS-CoV-2 HR2P (40 μM) at 37 °C for 1 h. HR1P was then added and incubated at 37 °C for 0.5 h. The mixture was loaded on a Tris-glycine gel (18%) for 3 h and visualized by Coomassie blue [17].

### 2.11. Structure Modeling

The predicted structure of 5-Helix was modeled using SWISS-MODEL software based on the homology of the 6-HB crystal structure (PDB: 6LXT) [7,18]. Figures were generated using the Pymol program.

### 2.12. Statistical Analysis

Statistical analysis was conducted by GraphPad Prism 9.0. Statistical significance of obtained data between different groups was established using Student’s unpaired two-tailed *t*-test. *p*-value below 0.05 was considered significant; * *p* < 0.05, ** *p* < 0.01, *** *p* < 0.001 and **** *p* < 0.0001.

## 3. Results

### 3.1. Design and Characterization of SARS-CoV-2 Spike 5-Helix

Several previous studies have identified HR1-targeting SARS-CoV-2 fusion inhibitory peptides or small molecule agents, such as EK1 [5], EK1C4 [7], IPB02 [19] and Salvianolic acid C [20]. In this study, we designed and constructed a 5-Helix fusion protein consisting of three copies of HR1P (a 44-mer peptide corresponding to residues L922~Q965 of HR1 in S2 of SARS-CoV-2) and two copies of HR2P (a 40-mer peptide corresponding to residues D1165~G1204 of HR2 in S2 of SARS-CoV-2). We also constructed flexible linkers (GGSGG) between HR1P and HR2P and (GSSGG) between HR2P and HR1P, as well as a His_6_-tag at the C-terminus (Figure 1A). We also synthesized peptides HR1P and HR2P, which can form a 6-HB fusion core when both peptides are mixed together (Figure 1B) [21]. The 5-Helix fusion protein is expected to form 5-Helix-bundle (5-HB) [22], with a structure similar to that of 6-HB but lacking one copy of HR2P. This results in the exposure of one hydrophobic groove that can be accessed and bound by an HR2P peptide or the HR2 region in the S2 subunit of SARS-CoV-2 (Figure 1B).

The expressed and purified 5-Helix protein showed a single band of about 24 kDa in SDS-PAGE (Figure 1C), which is consistent with its theoretical molecular weight. Results of Western blotting (WB) with an anti-His_6_ monoclonal antibody indicated that the protein migrated to the same position as that in SDS-PAGE (Figure 1C). The secondary structure of 5-Helix was investigated by CD spectroscopy. As shown in Figure 1D, 5-Helix displayed extremely high α-helicity (almost 100%), which is in agreement with its predicted structure. The melting temperature (*T_m_*) of 5-Helix was 51.71 °C, indicating its thermostability (Figure 1E) [12].

### 3.2. 5-Helix Bound HR2P with High Affinity

**Figure 2:**
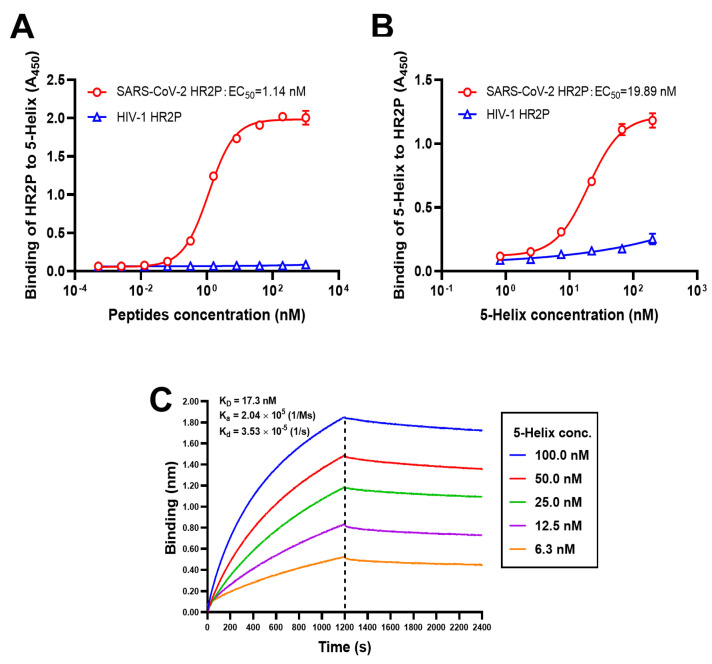
High binding affinity between 5-Helix and SARS-CoV-2 HR2P. Binding of 5-Helix to SARS-CoV-2 HR2P (**A**) and binding of SARS-CoV-2 HR2P to 5-Helix (**B**), as measured by ELISA. HIV-1 HR2P was used as negative control. Experiments were conducted in triplicate, and data are expressed as means ± SD. (**C**) Binding affinity between 5-Helix and SARS-CoV-2 HR2P was measured using biolayer interferometry (BLI).

Given that 5-Helix possesses high helical content and thermostability, we assessed the capacity of SARS-CoV-2 HR2P to bind it using a bidirectional ELISA. First, serially diluted SARS-CoV-2 HR2P was added to wells of a microplate coated with 5-Helix. The mean 50% effective concentration (EC_50_) of binding was 1.14 nM (Figure 2A). Second, serially diluted 5-Helix was added to wells of a microplate indirectly coated with SARS-CoV-2 HR2P, and the binding EC_50_ was 19.89 nM (Figure 2B). No significant interaction occurred between 5-Helix and HIV-1 HR2P (as a negative control). To quantitatively validate their binding affinity, biolayer interferometry (BLI) assay to determine the interactions between 5-Helix at five different concentrations and biotinylated SARS-CoV-2 HR2P was conducted. We found that 5-Helix bound SARS-CoV-2 HR2P with high affinity (K_D_ = 17.3 nM) and that the association and dissociation rate constant was 2.04 × 10^5^ Ms^−1^ and 3.53 × 10^−5^ s^−1^, respectively (Figure 2C). These findings suggest that the binding between 5-Helix and SARS-CoV-2 HR2P is specific and strong, thus establishing a firm foundation for disrupting SARS-CoV-2 6-HB formation.

### 3.3. 5-Helix Blocked Viral 6-HB Formation between HR1P and HR2P of SARS-CoV-2

**Figure 3:**
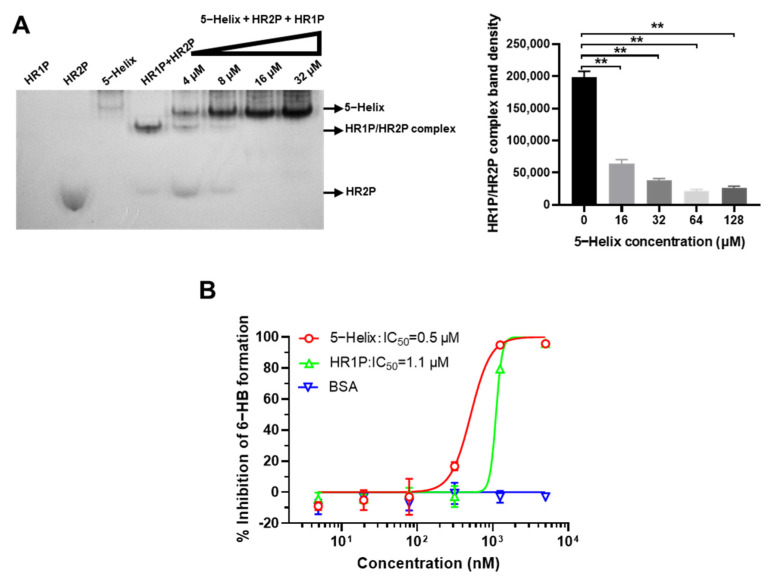
Disruption of 5-Helix on SARS-CoV-2 HR1–HR2 interaction. (**A**) 5-Helix-mediated disruption of 6-HB formation between SARS-CoV-2 HR1 and HR2 peptides as shown by N-PAGE. The 5-Helix was preincubated with SARS-CoV-2 HR2P before addition of HR1P. The total mixture was separated by N-PAGE. The intensity of HR1P/HR2P complex band (corresponding to the band of 6-HB) decreased with the increasing concentration of 5-Helix added. (**B**) 5-Helix-mediated inhibition of 6-HB formation between SARS-CoV-2 HR1 and HR2 peptides as shown by ELISA. Three independent experiments were performed, and data are shown as means ± SD, **, *p* < 0.01.

In order to further examine whether 5-Helix could competitively interact with SARS-CoV-2 HR2 to form 6-HB and block the formation between SARS-CoV-2 HR1P and HR2P, we performed Native PAGE by loading the mixtures of SARS-CoV-2 HR2P and 5-Helix at 16, 32, 64 and 128 μM in PBS to the gel before electrophoresis. As shown in Figure 3A, HR1P with net positive charge migrated upward and off the gel, whereas HR2P with net negative charge migrated downward and showed a band in the lower part of the gel. The density of the HR1P/HR2P complex (corresponding to the 6-HB fusion core) band decreased with increasing amounts of 5-Helix in a dose-dependent manner. Meanwhile, the density of the free HR2P band in the lower part of the gel also decreased with an increasing amount of 5-Helix, indicating that 5-Helix can interact with HR2P to form 6-HB and block 6-HB formation between HR1P and HR2P peptides. We then performed a competitive ELISA and found that 5-Helix could interact with HR2P, thus blocking HR2P binding to the coated HR1P with an IC_50_ of 0.5 μM, while that of free HR1P for blocking 6-HB formation was 1.1 μM. However, the unrelated protein BSA had no effect on blocking 6-HB formation (Figure 3B). These results suggest that 5-Helix can effectively compete with viral HR1 to bind viral HR2 for blocking viral HR1–HR2 interaction to form the 6-HB fusion core.

### 3.4. 5-Helix Inhibited SARS-CoV-2 S-Mediated Membrane Fusion and PsV Infection

**Figure 4:**
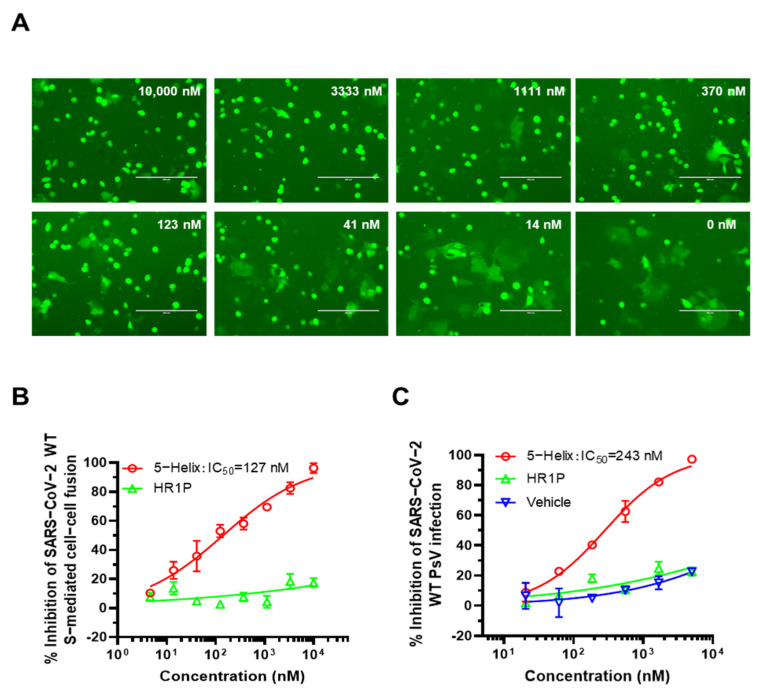
Inhibitory activity of 5-Helix against its S-mediated cell–cell fusion and SARS-CoV-2 PsV infection. (**A**) Fluorescence images of SARS-CoV-2 S-mediated cell–cell fusion in the presence of 5-Helix with different concentrations. Scale bars, 200 μm. The percentage of fused cells was calculated. (**B**) Statistical analysis of 5-Helix inhibitory activity on cell–cell fusion. Three independent experiments were performed, and data were presented as means ± SD. (**C**) Inhibitory activity of 5-Helix, SARS-CoV-2 HR1P and protein vehicle on SARS-CoV-2 WT (D614G) PsV infection. Each sample was tested in triplicate, and data are shown as means ± standard deviations (SD). Three independent experiments were performed, and similar results were obtained.

We next measured the inhibitory activity of 5-Helix against SARS-CoV-2 S-mediated cell–cell fusion. As shown in Figure 4A,B, 5-Helix effectively inhibited SARS-CoV-2 S-mediated cell–cell fusion in a dose-dependent manner with a mean IC_50_ value of 128 nM, while HR1P peptide showed no significant inhibition of cell–cell fusion at the concentration as high as 10,000 nM. Then, we tested the inhibitory activity of 5-Helix against SARS-CoV-2 PsV infection. As shown in Figure 4C, 5-Helix effectively inhibited SARS-CoV-2 PsV infection in Caco-2 cells with a mean IC_50_ value of 243 nM, while, again, HR1P showed no remarkable inhibitory activity at the concentration up to 5000 nM. The inhibitory activity of 5-Helix on SARS-CoV-2 S-mediated cell–cell fusion and PsV infection is comparable to that of the pan-CoV fusion inhibitor EK1 [5]. 

### 3.5. 5-Helix Showed Stable Antiviral Activity against Pseudotyped SARS-CoV-2 Variants

**Figure 5:**
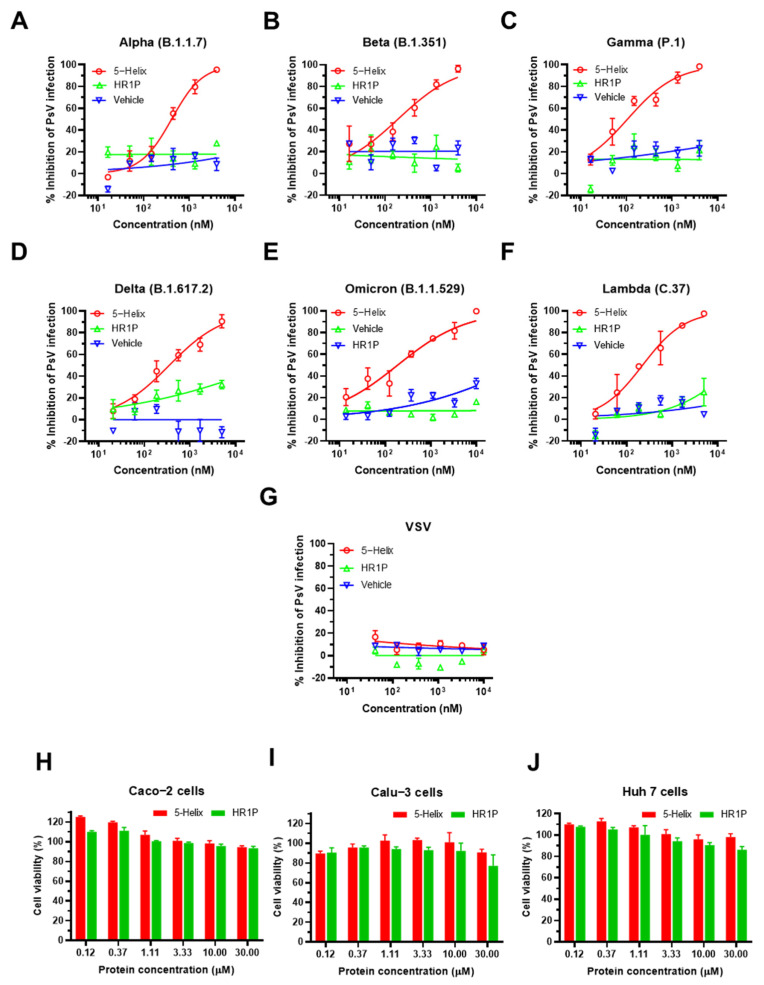
5-Helix-mediated inhibition of infection by pseudotyped SARS-CoV-2 variants and its cytotoxicity. Inhibitory activity of 5-Helix on infection of five VOC (Alpha, Beta, Gamma, Delta, Omicron) PsVs and one VOI (Lambda) PsV in Caco-2 cells (**A**–**F**) with VSV PsV as negative control (**G**). The cytotoxicity of 5-Helix and HR1P on Huh 7 cells, Caco-2 cells and Calu-3 cells were tested using CCK-8 assay (**H**–**J**). Each sample was tested in triplicate, and three experiments were repeated. Data are shown as means ± SD.

Both VOC and VOI have different degrees of resistance to neutralizing antibodies and the resultant reduction in vaccine efficacy [23,24,25,26,27]. In order to address these differences, the inhibitory activity of 5-Helix was determined against changes in the infection properties of SARS-CoV-2 variants for five previously constructed VOC PsVs, including the Omicron PsV and one VOI (Lambda, C.37) PsV. We found that 5-Helix exhibited potent inhibitory activity against infection by Alpha, Beta, Gamma, Delta and Lambda PsVs with IC_50_ of 364, 170, 142, 369 and 238 nM, respectively, whereas HR1P peptide showed no obvious inhibition (Figure 5A–D,F). Surprisingly, 5-Helix displayed the highest inhibitory activity against Omicron PsV infection with IC_50_ of 141 nM (Figure 5E), even while Omicron had the highest resistance to SARS-CoV-2 neutralizing antibodies [28]. However, 5-Helix showed no significant inhibitory effect on control VSV PsV infection at concentrations up to 10,000 nM (Figure 5G). We wanted to rule out any potential cytotoxicity of 5-Helix that would result in decreased infectivity of the PsVs to target cells. Therefore, we tested the cytotoxicity of 5-Helix on human hepatocellular carcinoma cell line Huh 7, human colon carcinoma cell line Caco-2 and human lung adenocarcinoma cell line Calu-3 using CCK8 assay. Similar to the HR1P peptide, 5-Helix at the concentration as high as 30 μM showed no significant cytotoxicity (Figure 5H–J) and had a selectivity index (SI) over 80 (Table 1), indicating that 5-Helix is a stable inhibitor to combat the continuously emerging variants of SARS-CoV-2 with extremely low toxic effect in vitro.

### 3.6. 5-Helix Exhibited Inhibitory Activity against Both Authentic SARS-CoV-2 WT and Delta Variant

**Figure 6:**
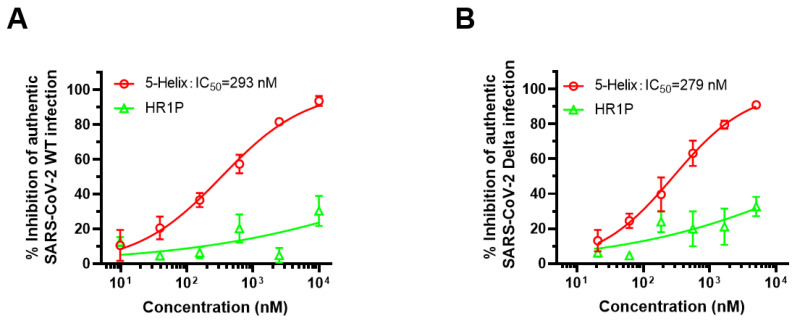
5-Helix-mediated inhibition of the authentic SARS-CoV-2 WT (nCoV-SH01) strain and Delta variant. The inhibitory activity of 5-Helix on infection of the authentic SARS-CoV-2 WT (nCoV-SH01) (**A**) and Delta variant (**B**) in Calu-3 cells was evaluated by RT-qPCR assay. Three experiments were performed, and the data are shown as means ± SD. SARS-CoV-2 HR1P served as negative control.

Subsequently, we evaluated the inhibitory activity of 5-Helix on infection by the authentic SARS-CoV-2 WT (nCoV-SH01) strain and its Delta variant in Calu-3 cells. We found that 5-Helix was equally effective against authentic SARS-CoV-2 WT (nCoV-SH01) strain and Delta variant infection with mean IC_50_ of 293 nM (Figure 6A) and 279 nM (Figure 6B), respectively. These results suggest that 5-Helix has similar inhibitory activity against the authentic WT strain and Delta variant infection in vitro.

## 4. Discussion

During the current COVID-19 pandemic with the continuous emergence of VOCs and VOIs, the development of highly effective and broader-spectrum antivirals against SARS-CoV-2 and persistently emerging SARS-CoV-2 variants is urgently needed. To this end, viral fusion/inhibitors targeting the functional domains in the S2 subunit in the S protein of SARS-CoV-2, e.g., HR1 and HR2 domains in the S2 subunit, are more attractive than those targeting the S1 subunit, e.g., the RBD and NTD. Thus far, these have been the most important target sites for the development of neutralizing antibody therapeutics and vaccines. However, the functional domains (e.g., RBD) in the S1 subunit are prone to natural mutation because their surfaces in the native conformation are generally exposed to the host immune system, especially antibodies. That is why the Omicron variant with its multiple mutations in RBD is so resistant to most neutralizing antibodies targeting the RBD. In contrast, most parts of the S2 subunit are shielded by the S1 subunit in native conformation and, hence, neither accessible to nor recognized by antibodies, in most cases, with less chance for mutation. HR1 and HR2 domains in the S2 subunit are only transiently exposed for a few minutes immediately after binding between the RBD in S1 and the target cell’s ACE2 receptor. Even at the fusion intermediate state, HR1 and HR2 domains still cannot be accessed by antibody IgG with a molecular weight of 150 kDa because of the constrained space of NHR-trimer in the fusion intermediate state, which has a steric exclusion of molecules with >40 kDa [29]. Accordingly, the HR1 and HR2 domains are the most conserved regions in coronavirus S proteins; consequently, antivirals targeting HR1 and HR2 domains are expected to have high genetic barriers to resistance.

We previously identified a series of peptide-based fusion/entry inhibitors targeting the HR1 domain of HIV-1 (e.g., SJ-2176) [30], SARS-CoV (SC-1) [31], MERS-CoV (MERS-HR2P) [32], HCoV-OC43 (OC43-HR2P) [5] and SARS-CoV-2 (2019-nCoV-HR2P) [6], as well as to pan-CoV, including EK1 [5], EK1C4 [7,33] and EKL1C [8]. Notwithstanding these advances, HR2 should be a better target for the development of broad-spectrum HCoV fusion/entry inhibitors since the amino acid sequence of the HR2 domain is more conserved than that of HR1. For example, the amino acid sequences of HR1 and HR2 domains of SARS-CoV-2 are 92.6% and 100% identical to those of HR1 and HR2 of SARS-CoV, respectively [6]. However, peptides derived from the SARS-CoV-2 HR1 domain, similar to those derived from the HIV-1 HR1 domain, have no, or very low, antiviral activity when compared with the corresponding HR2 peptides. Therefore, compared to HR2, HR1 peptides with more hydrophilic amino acids tend to aggregate in physiological solution in order to form a stable HR1-trimer [6,34]. 

We previously used sequences of the HIV-1 gp41 HR1 domain and the N terminus of Foldon (Fd), the natural trimerization domain of T4 bacteriophage fibritin, to construct plasmids encoding the recombinant proteins N36Fd and N28Fd. We found that they could form stable HR1-trimers and interact with the HIV-1 gp41 HR2-peptide to form soluble 6-HB and that they had more potent inhibitory activity than T20 against HIV-1 infection [34]. However, this kind of recombinant protein, when used in HIV-1-infected patients for a long time, may induce antibodies against Fd to suppress the antiviral activity of N36Fd and N28Fd. Although 5-Helix, as a recombinant protein, may also elicit antibodies after repeated administration in COVID-19 patients. However, different from the anti-HIV therapeutics that have to be repeatedly used for a long time, an antiviral against SARS-CoV-2 infection must be only used at the early stage of infection for a few days while the antibodies specific for the antiviral may be elicited after the stop of its use. Therefore, the induction of antibodies against 5-Helix may not be an issue for its future clinical application.

Root et al. [22] applied another approach to develop an engineered HIV-1 fusion/entry inhibitory protein consisting of three NHR-helices and two CHR-helices of the HIV-1 gp41. Similar to N36Fd, this engineered protein could also bind with the CHR-peptide of the HIV-1 gp41 to form 6-HB or interact with the viral gp41 CHR region to block the formation of the viral gp41 fusion core, thus inhibiting HIV-1 infection. Here, we used a similar approach to construct the engineered protein 5-Helix comprised of three HR1 and two HR2 segments of the SARS-CoV-2 S protein S2 subunit. We found that 5-Helix displayed a helical structure with a high α-helicity and *T_m_* value of ~52 °C. It could bind with SARS-CoV-2 HR2P peptide with high affinity (K_D_ = 17.3 nM) and block 6-HB formation between the HR1 and HR2 peptides of SARS-CoV-2. The 5-Helix was highly effective against SARS-CoV-2 S-mediated cell–cell fusion, and it inhibited infection of the pseudotyped SARS-CoV-2 WT (D614G) and five VOCs, including Alpha, Beta, Gamma, Delta and Omicron variants and one VOI (Lambda variant). It was almost equally potent against the authentic SARS-CoV-2 WT (nCoV-SH01) strain and Delta variant infection. 

EK1 [5], a pan-CoV fusion inhibitor developed by our group, showed inhibitory activity against infection of SARS-CoV-2 and VOC PsVs with IC_50s_ in a range of 119~2000 nM, while that of 5-Helix ranging from 140 nM to 369 nM. In general, 5-Helix exhibits about two- to three-fold higher antiviral activity against SARS-CoV-2 and VOCs than EK1. The lipopeptides EK1C4 [7] and EKL1C [8] are more potent than 5-Helix against infection of SARS-CoV-2 and VOCs. However, the lower solubility and higher production cost of these lipopeptides than EK1 peptide may limit their further development, while 5-Helix may have a lower production cost and longer half-life because it can be produced on a large scale in yeast or *E. coli* expression systems and have bigger molecular size. Moreover, all these peptide- and lipopeptide-based pan-CoV fusion inhibitors target the HR1 domain, while 5-Helix targets the more conserved HR2 domain (Figure S1) [6]. Therefore, 5-Helix may have a higher genetic barrier to resistance than the EK1 peptide and lipopeptides. All these results suggest that 5-Helix is a highly promising candidate for development as a novel prophylactic or therapeutic for prevention or treatment of infection by currently circulating and future emerging SARS-CoV-2 variants.

## Figures and Tables

**Table 1:** Inhibitory activity of 5-Helix against SARS-CoV-2 PsV infection and the selectivity index (SI).

Pseudovirus	IC_50_ (nM)	CC_50_ (μM) *	Selectivity Index (SI)
WT (D614G)	243.44 ± 6.28	>30	>100
Alpha (B.1.1.7)	363.67 ± 3.77	>30	>80
Beta (B.1.351)	169.67 ± 4.88	>30	>100
Gamma (P.1)	141.78 ± 1.32	>30	>200
Delta (B.1.617.2)	368.91 ± 9.24	>30	>80
Omicron (B.1.1.529)	140.73 ± 1.26	>30	>200
Lambda (C.37)	238.25 ± 5.57	>30	>100

* CC_50_, the half-cytotoxic concentration of 5-Helix on Huh 7, Caco-2 and Calu-3 cells tested.

## Data Availability

Not applicable.

## Supplementary Materials

The following supporting information can be downloaded at: https://www.mdpi.com/article/10.3390/v14030597/s1, Figure S1. Sequence alignment of HR1 and HR2 domains in the SARS-CoV-2 wildtype strain and its variants.
