# Supplementary material for: A Five-Helix-Based SARS-CoV-2 Fusion Inhibitor Targeting Heptad Repeat 2 Domain against SARS-CoV-2 and Its Variants of Concern
Source: Viruses. 2022 Mar 13;14(3):597. doi: 10.3390/v14030597 (PMC8955665; doi:10.3390/v14030597)
Supplement: Supplementary file 1 [file viruses-14-00597-s001.zip › viruses-1599440-supplementary.pdf]

viruses

MDPI

Article

A five-helix-based SARS-CoV-2 fusion inhibitor targeting heptad repeat 2 domain against SARS-CoV-2 and its variants of concern

Lixiao Xing [1,†], Xinfeng Xu [2,†], Wei Xu [1,†], Zezhong Liu [1], Xin Shen [1], Jie Zhou [1], Ling Xu [1], Jing Pu [1], Chan Yang [2], Yuan Huang [2], Lu Lu [1,*], Shibo Jiang [1,*] and Shuwen Liu [2,*]

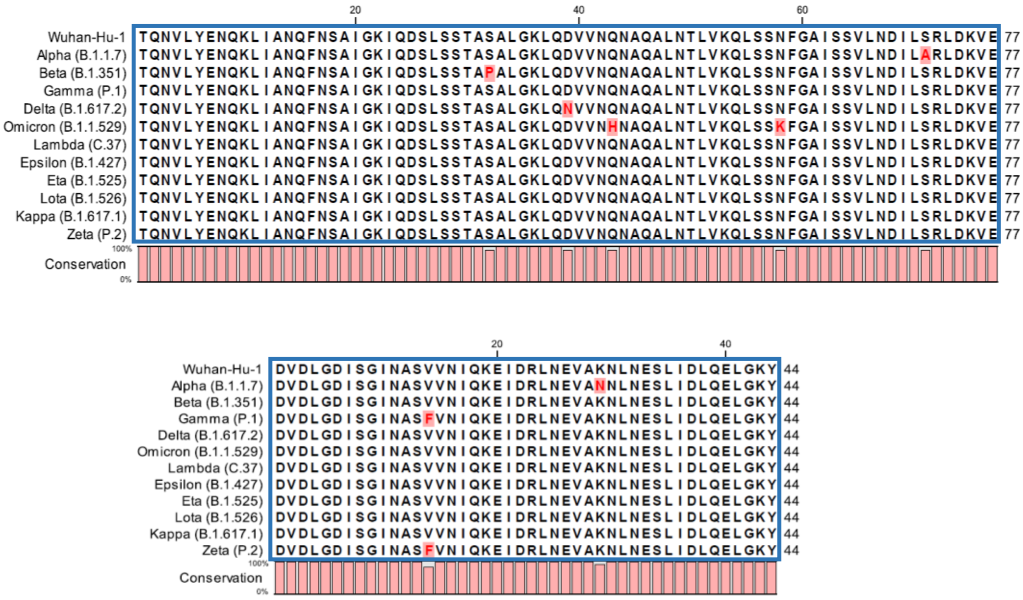

Figure S1. Sequence alignment of HR1 and HR2 domains in the SARS-CoV-2 wildtype strain and its variants. The sequence comparison of the HR1 and HR2 domains in SARS-CoV-2 wildtype (Wuhan-Hu-1, GenBank: NC_045512), Alpha (GenBank: OM757358.1), Beta (GenBank: OM736179.1), Gamma (GenBank: OM146072.1), Delta (GenBank: OM754056.1), Omicron (GenBank: OM757978.1), Lambda (GenBank: OM486769.1), Epsilon (GenBank: OM757360.1), Eta (GenBank: OM720711.1), Lota (GenBank: OM516760.1), Kappa (GenBank: OM680927.1), and Zeta (GenBank: OM485821.1). The different amino acids in the SARS-CoV-2 variants, compared to the SARS-CoV-2 wildtype strain, are highlighted in red color and pink shade. The amino acid conservation is indicated by the red bars.
